# Application of MOX Sensors to Determine the Emission of Volatile Compounds in Corn Groats as a Function of Vertical Pressure in the Silo and Moisture Content of the Bed

**DOI:** 10.3390/s24072187

**Published:** 2024-03-28

**Authors:** Robert Rusinek, Aleksandra Żytek, Mateusz Stasiak, Joanna Wiącek, Marek Gancarz

**Affiliations:** 1 Institute of Agrophysics Polish Academy of Sciences, Doświadczalna 4, 20-290 Lublin, Polandj.wiacek@ipan.lublin.pl (J.W.); marek.gancarz@urk.edu.pl (M.G.); 2 Faculty of Production and Power Engineering, University of Agriculture in Krakow, Balicka 116B, 30-149 Krakow, Poland

**Keywords:** corn groats, cereal moisture, grain storage, volatile compound sensors, electronic nose, GC–MS, chemometrics

## Abstract

This study was focused on the analysis of the emission of volatile compounds as an indicator of changes in the quality degradation of corn groats with 14% and 17% moisture content (wet basis) using an electronic nose (Agrinose) at changing vertical pressure values. The corn groats were used in this study in an unconsolidated state of 0 kPa (the upper free layer of bulk material in the silo) and under a consolidation pressure of 40 kPa (approximately 3 m from the upper layer towards the bottom of the silo) and 80 kPa (approximately 6 m from the upper layer towards the bottom of the silo). The consolidation pressures corresponded to the vertical pressures acting on the layers of the bulk material bed in medium-slender and low silos. Chromatographic determinations of volatile organic compounds were performed as reference tests. The investigations confirmed the correlation of the electronic nose response with the quality degradation of the groats as a function of storage time. An important conclusion supported by the research results is that, based on the determined levels of intensity of volatile compound emission, the electronic nose is able to distinguish the individual layers of the bulk material bed undergoing different degrees of quality degradation.

## 1. Introduction

Corn groats are a product of grinding corn kernels. They are very often used as a basis for the production of extrudates, mainly snacks for children [1,2]. In addition to their high content of nutrients, vitamins, and fibre, corn groats are a rich source of vitamin A.

One of the technological processes applied to corn groats in order to ensure the continuity of production of groat-based food is storage in tanks and silos. Improper storage of cereal products, including corn groats, may lead to the formation of mycotoxins in food, which have an adverse effect on the consumer’s organism. The main toxins produced by pathogens are zearalenone (with estrogenic activity; produced by *Fusarium*) [3], aflatoxins (produced by *Aspergillus flavus*; they may appear already during vegetation in the field, but their highest concentration is associated with improper storage of the materials [4,5,6]), fumonisins (with carcinogenic activity; produced by *Fusarium*) [7,8], and ochratoxin A (produced by *Penicilium verrucosum* in improper drying and storage conditions [9,10].

A cereal product with a higher fragmentation degree has a greater tendency to enhance the intensity of biochemical and chemical transformations than uncrushed kernels due to the destruction of the natural system of enzymes and their substrates in the grain. Corn groats have a larger specific surface area than the whole grain, hence their higher susceptibility to oxidation. One of the first symptoms of the degradation of groats is an increase in acidity caused by lipase activity [11]. 

It is important to prevent the formation and development of mycotoxins in cereals, especially those intended for consumption [12]. There are two methods for preventing the phenomenon: detoxification and monitoring of storage conditions. Moreover, grain or groat beds should be stored at an appropriate temperature and moisture (w.b.—wet basis). The moisture content in corn products has been defined by the special PN-A-74205 standard and should not exceed 14.5% [13,14]. Additionally, processing the raw material can substantially reduce the amount of toxins in food [15]. 

The electronic nose offers potential as a rapid and cost-effective portable device that allows for quick screening in terms of determining the physicochemical state of plant materials. There are a dozen technical solutions regarding sensors used in electronic noses. In this work, the authors focused on the use of MOX (metal oxide semiconductor) sensors [16,17,18]. 

The aim of this study was to analyze the possibility of detection of unfavorable quality changes occurring in the corn groats bed with the use of an electronic nose with MOX sensors [19]. The present analyses were carried out over a nine-day storage period. The deterioration of the quality of corn groats was detected by analysis of changes in the intensity of emission of volatile compounds under different consolidation pressures corresponding to natural storage conditions in industrial silos (0, 40, and 80 kPa) at storage moisture of 14% (w.b.) and elevated bed moisture of 17% (w.b.). Although storage-related studies considering the storage time have almost sufficiently been reported, there are no literature data on the use of an electronic nose to study quality degradation as a function of consolidation pressure corresponding to the natural pressure distribution along the vertical axis in silos. This is particularly important, as bulk materials, including corn groats, are subjected to vertical pressure during storage in tanks and silos, which increases along the distance from the upper free layer of the bed. Higher consolidation pressure leads to caking of the material, poorer moisture removal and bed ventilation, and the formation of self-heating spots, which directly results in the acceleration of quality degradation processes [20]. 

In connection with the above information, the authors formulated the following research hypotheses:

**H1**:  *The emission level of volatile compounds depends not only on the humidity of the bulk material and storage time but also on the density of the bulk material, which is influenced by the vertical consolidation pressure in the silo;*

**H2**:  *Electronic nose based on MOX sensors is a precise tool that is able to distinguish in real-time areas in a homogeneous bulk material with different levels of volatile compound emissions.*

In the present study, chromatographic techniques for the detection of volatile compounds were used as reference tests to verify the results of the electronic nose-based determination of the level of emission [19].

## 2. Materials and Methods

### 2.1. Materials

The corn groats used in the analyses were purchased from a commercial store. The product was characterized by a 14% moisture content, corn-specific flavor and aroma, a yellow color, and a fat content of 0.89%. The particle size was in the range of 700–300 μm. No mineral or metal contamination or pest infestation was detected. 

The corn groats were used in this study in an unconsolidated state of 0 kPa (bulk density) and under consolidation pressures of 40 and 80 kPa, respectively, corresponding to pressures prevailing in industrial silos (0 kPa—upper free layer of bulk material; 40 kPa—approximately 3 m below the upper free layer of bulk material; and 80 kPa—approximately 6 m below the upper free layer of bulk material; approximate calculations for medium-slender and low silos) [21,22]. 

The Instron 8872 testing machine (825 University Ave, Norwood, MA, 02062-2643, USA) was used to consolidate the material in cylinders equipped with VOC sampling valves. The groats had standard (14%) and increased (17%) moisture content. To accelerate fungal flora growth in the raw material, the cylinders filled with corn were placed in containers at an elevated temperature of 32 °C. The analyses were performed during the 9-day storage of the material until the first signs of quality degradation were visible.

### 2.2. Gas Chromatography–Mass Spectrometry Analysis

The GC–MS analyses of the samples were performed using a Trace GC Ultra gas spectrometer coupled to an ITQ 1100 mass spectrometer from ThermoFisher Scientific (Waltham, MA, USA) following the procedure described in our previous publications. An SPME (solid-phase micro-extraction) fiber with an absorbent (50/30 μm Divinylbenzene/Carboxene/Polydimethylsiloxane (DVB/CAR/PDMS), Stableflex (2 cm) 24 Ga (Sigma Aldrich, Poznań, Poland), was placed in the measurement chamber for 30 min together with the material emitting volatile compounds and then transferred to a GC injector for 5 min to desorb VOCs. The chromatographic analysis was carried out with the use of a Zebron ZB-5Msplus Capillary GC 30 m × 0.25 mm × 0.25 um column. The compounds were identified using the Wiley library. Compounds with a 75% identity level were selected [23]. 

### 2.3. An Electronic Nose

An Agrinose was used to analyze the intensity of volatile compound emissions as a function of consolidation pressure, bed moisture, and storage time. This electronic nose, based on a matrix of MOX sensors (also called MOS sensors), was constructed at the Institute of Agrophysics in Lublin. In previous studies, it was used for assessments of, e.g., the degradation of rapeseed beds, the degree of rapeseed oil degradation as a function of frying temperature, the shelf life of bread supplemented with various plant fibers, the effects of coffee roasting, and many other phenomena. The parameters of the electronic nose were discussed in detail in previous publications [24] and are presented in the Supplementary Materials (01_Supplementary_Materials. Agrinose).

### 2.4. Statistical Analysis

Statistica software (version 12.0, StatSoft Inc., Tulsa, OK, USA) was used for statistical analyses. Principal component analysis (PCA), analysis of variance, and determination of correlations were performed at a significance level of α = 0.05. The PCA data matrix for the statistical analysis of the results of tests had 18 columns (volatile compounds, volatile emission intensity) and 27 rows (days of storage) [19] for 14% moisture content and the same for 17% moisture content. The input matrix was scaled automatically. The optimal number of principal components obtained in the analysis was determined based on the Cattel criterion [24].

The proposed analysis of the principal components is intended to describe the differences between the test results for corn groats with the storage moisture content recommended by the standards (14% w.b.) and the case of abnormal moisture content significantly exceeding the applicable standards (17% w.b.). 

## 3. Results and Discussion

### 3.1. GC–MS and Electronic Nose Analysis

The chromatographic analyses revealed the presence of several main volatile organic compounds in each variant of consolidation pressure and moisture [25]. The “02_Supplementary_materials_Volatile_compounds_” file contains detailed data obtained in this study. The volatile compounds were used as a reference for the determination of the intensity of VOC emission (volatile organic compounds) using the electronic nose. The main volatile compounds were classified into the groups of alcohols, ketones, azines, esters, acids, terpenes, and hydrocarbons, and their percentage share was calculated (Table 1) [25].

Compounds that were not assigned to the above-mentioned groups were marked as “others”. As shown in Table 2, regardless of the moisture level and consolidation pressure, alcohols, acids, ketones, esters, and hydrocarbons were detected in all cases. No azines were detected in the 14% w.b. group or in the 40 and 80 kPa variants. In turn, terpenes were not found in the 14% w.b. samples and in the 0 and 80 kPa variants. Alcohols were the largest group of volatile compounds in all cases, especially in the 17% moisture group.

The results of the electronic nose determinations are presented in the “03_Supplementary_materials_An_electornic_nose_results” file. They indicate that three of the eight sensors exhibited the highest sensitivity to the emission of volatile compounds, i.e., TGS-2602 (ammonia, hydrogen sulfide; high sensitivity to VOCs and odorous gases), TGS-2620 (solvent vapors, volatile vapors, alcohol), and TGS-2600 (general air contaminants, hydrogen, and carbon monoxide). Probably, the significant share of the alcohol group in the individual corn groats samples had a significant impact on the sensitivity of these sensors, especially in the 17% w.b. variant.

### 3.2. Principal Component Analysis (PCA)

**Figure 1:**
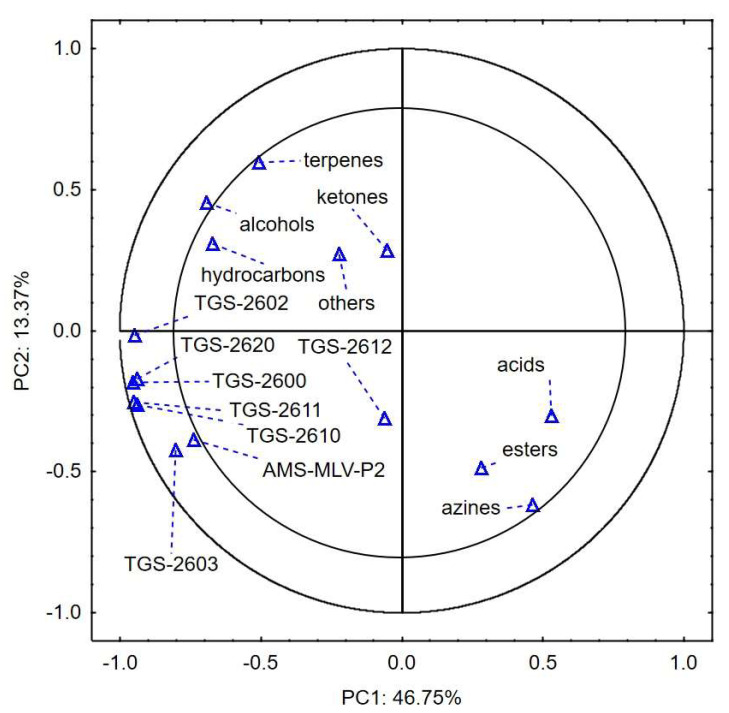
Loading plot of the principal components analysis of seven chemical groups detected in corn groats at 14% wet basis, consolidation of 0, 40, and 80 kPa, and eight sensors carried out for 9 days of corn groats storage.

**Figure 2:**
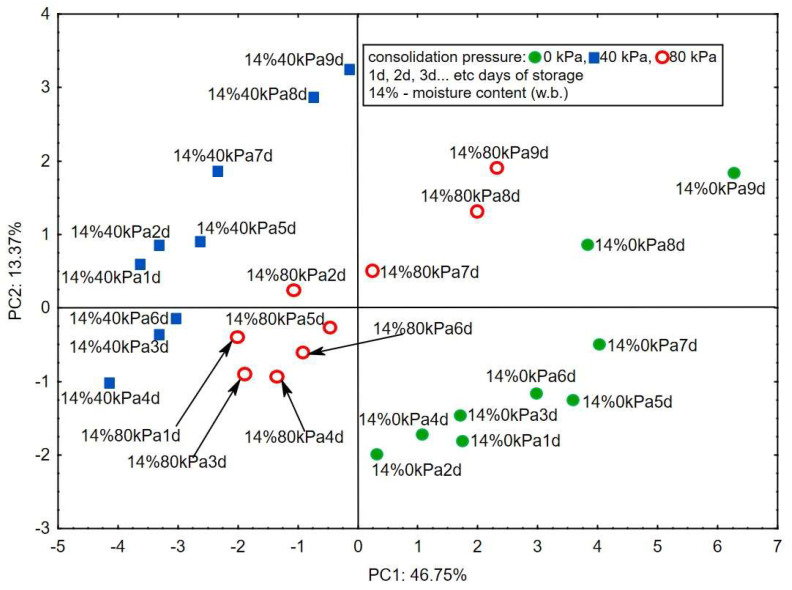
Score plot of the principal components analysis of seven chemical groups detected in corn groats at 14% wet basis, consolidation of 0, 40, and 80 kPa, and eight sensors carried out for 9 days of corn groats storage.

Figure 1 present the projection of the variables in the different variants of consolidation and moisture of corn groats. The principal components explain the correlations between the compounds identified during the analysis and the sensor responses [19]. Figure 2 show changes in the VOC emissions correlated with the responses of the e-nose sensors during storage at consolidation pressures of 0, 40, and 80 kPa. 

Figure 1 shows the projection of the variables for the 14% moisture content and 0, 40, and 80 kPa consolidation on the PC1 (46.75%) and PC2 (13.37%) planes. The first two principal components, PC1 and PC2, explain 60.12% of the variability of the system. A strong positive correlation was observed between the sensor responses related to the intensity of VOC emission and the changes in the content of alcohols, hydrocarbons, and terpenes. There was a negative correlation between the sensor responses and the content of acids, esters, and azines. The groups of ketones and “others” did not correlate with these variables; likewise, the responses of sensor TGS-2612 did not detect the changes occurring in the samples during storage. The values of the aforementioned variables are distributed in the closest vicinity of PC1 = 0 and PC2 = 0.

In Figure 2, the PC1 and PC2 describe the degree of quality degradation of the corn groats progressing over 9 days of storage at 46.75% and 13.27%, respectively. Similar results were reported in studies on rapeseed and corn grains [23,26], but the quality degradation was described only by PC1. The research results presented in Figure 2 are similar to those obtained by Wang et al. (2013) [27]. The authors examined the stages of corn infection with mold using an electronic nose with MOX sensors. The analysis of the main components by these authors showed a similar tendency, which can be seen in Figure 2 for maize groats. In terms of the presentation of quality degradation as a function of storage time, the electronic nose turned out to be a useful tool for monitoring the biological state of agricultural raw materials. Similar results were obtained for corn grain by Falasconi et al. already in 2005 [28] and next in 2011 [29]. The authors demonstrated in their work the capability of the electronic nose to detect fungal contamination of maize, mainly by *Fusarium*. However, the present study was focused on the determination of a potential correlation with not only the storage time but also the consolidation pressure. In other words, the aim was to determine the variability in the emission of volatile compounds detected with the use of an electronic nose along the vertical axis of the individual sectors of the corn groats-filled silo over the same storage time [16]. Figure 2 clearly shows three groups representing three different corn groat consolidation layers: 0, 40, and 80 kPa. In the 14% w.b. moisture variant, the 80 kPa group is located between the 0 kPa and 40 kPa groups, hence the conclusion that the electronic nose differentiates individual groups successfully [30]. However, in the case of the appropriate moisture recommended for storage by the relevant standards, there were no unfavorable phenomena that would differentiate the individual layers (groups) in relation to PC1. The recommended storage moisture level stabilizes and unifies the structure of the corn groats bed in its entire volume as a function of storage time [31]. 

**Figure 3:**
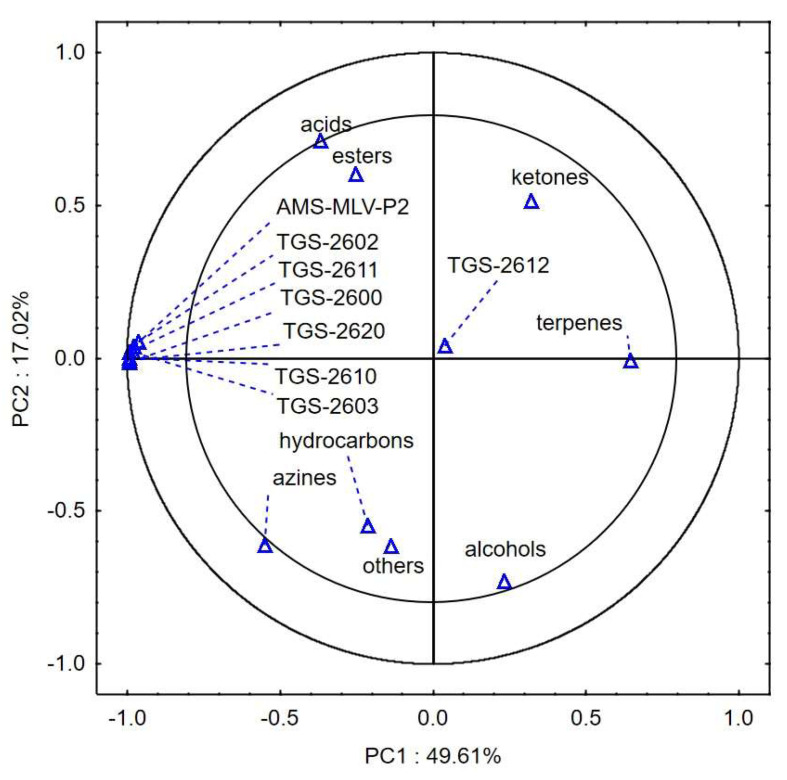
Loading plot of the principal components analysis of seven chemical groups detected in corn groats at 17% wet basis, consolidation of 0, 40, and 80 kPa, and eight sensors carried out for 9 days of corn groats storage.

Figure 3 shows the projection of the variables at 17% moisture content and 0, 40, and 80 kPa consolidation on the PC1 (49.61%) and PC2 (17.02%) planes. The first two principal components, PC1 and PC2, explain 66.63% of the variability of the system. A negative correlation was found between the sensor responses related to the intensity of VOC emission and the changes in the content of terpenes. There was also a negative correlation between the changes in the content of alcohols, azines, hydrocarbons, and other substances and the content of acids, ketones, and esters. As in the case of the 14% w.b. variant, sensor TGS-2612 did not detect the changes occurring in the stored samples. The location on the projection plane of the variables was almost PC1 = 0 and PC2 = 0.

**Figure 4:**
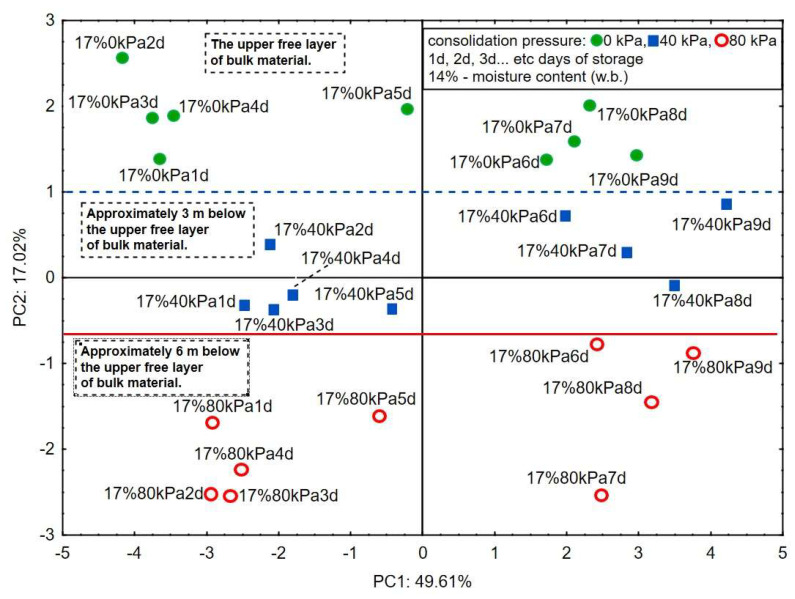
Score plot of the principal components analysis of seven chemical groups detected in corn groats at 17% wet basis, consolidation of 0, 40, and 80 kPa, and eight sensors carried out for 9 days of corn groats storage.

Figure 4 shows the projections of the cases on the PC1 and PC2 planes. The first principal component, PC1, describes the quality degradation of the corn groats taking place over nine storage days at all three consolidation pressure levels of 49.61%. In contrast to the 14% w.b. moisture variant, the second principal component, PC2, describes the changes in the VOC emission related to the height of the layer in the vertical axis of the silo of 17.02%. Taking into account that the loss of quality of bulk materials in silos may generate the effect of self-heating of the deposit, similar results of heterogeneity of quality changes in a model silo were obtained by Rusinek and Kobyłka (2014) [32]. 

The changes in the quality degradation as a function of time are described above the blue dashed line for the upper layer—0 kPa; between the blue and solid red line for the middle layer—40 kPa; and below the red line for the closest layer to the silo bottom—80 kPa. Probably, the clear differentiation between the three layers of the corn groats bed by the electronic nose in relation to PC2 is the result of the enhancement of the quality degradation processes with the increasing corn groats moisture [20,33] relative to the moisture level recommended by storage standards (moisture content not more than 14% w.b.) [34] and the selective sensitivity of MOX sensors. This observation is consistent with the generally known trend that the safe moisture content for long-term storage of corn and its semi-finished products must be lower than 14%. When this value is exceeded, the factor resulting from the amount of vertical pressure determines the acceleration of the deterioration effect of bulk materials stored in silos. This relationship in the case of 17% humidity is described by the second main component, PC2. 

The high sensitivity of metal oxide sensors to the emission of volatile substances from corn was also demonstrated by Machungo et al. (2022) [17]. Their results suggest that an electronic nose equipped with doped metal oxide semiconductor sensors and thermocycling is more effective for the detection of aflatoxin contamination of maize than conducting polymer sensors. A review by Janik et al. (2021), similarly to the research results presented in this work, puts the electronic nose on par with other precise instrumental methods as an important tool in the diagnostics of corn quality. This is important in light of the information that has flowed from storage practice that the uneven loss of the quality of bulk materials throughout the entire volume of silos is a common phenomenon that may depend on local changes in the moisture in the silo ecosystem, internal temperatures, and vertical loads increasing with the distance from the upper free layer towards the bottom.

## 4. Conclusions

The results of the determination of the intensity of VOC emission using the electronic nose contributed to the description of the corn groats quality degradation as a function of storage time.

In the case of sample moisture of 14% w.b., there was a strong positive correlation between the sensor responses related to the intensity of VOC emissions and the changes in the content of alcohols, hydrocarbons, and terpenes. A negative correlation was found between the sensor responses and the content of acids, esters, and azines. In the 17% w.b. moisture variant, a negative correlation was observed between the sensor responses to the intensity of VOC emission and the changes in the content of terpenes. Similarly, a negative correlation was found between the changes in the content of alcohols, azines, hydrocarbons, and other substances and the content of acids, ketones, and esters. The response of the TGS-2612 sensor to the changes in the VOC emission intensity was negligible. The sensor did not detect the changes in the quality degradation of the corn groats.

Bulk material stored in a silo undergoes qualitative degradation in a heterogeneous manner, as demonstrated by the analysis of volatile substances. The electronic nose equipped with MOX sensors can be a useful tool for differentiation of the level of quality degradation due to the specific emission of volatile compounds from each layer of the corn groats bed along the vertical axis of the silo, which was particularly well visible at the 17% moisture of the corn groats.

## Figures and Tables

**Table 1:** Average percentage share of groups of volatile organic compounds during 9-day corn groat storage.

Consolidation	Moisture Content	Days	Alcohols	Acids	Ketones	Esters	Hydrocarbons	Azines	Terpenes	Others
0 kPa	14%	1	8.59	5.84	17.53	50.86	9.62	3.44	n.d.	4.12
		2	10.73	19.17	21.58	35.50	8.10	2.65	n.d.	2.27
		3	5.91	31.73	22.40	30.26	5.61	1.58	n.d.	2.52
		4	5.35	34.03	21.24	30.74	3.90	1.24	n.d.	3.50
		5	5.30	9.85	10.98	66.67	n.d.	3.03	n.d.	4.17
		6	3.77	55.03	7.86	18.87	6.29	3.46	n.d.	4.72
		7	3.48	50.87	5.22	27.39	6.96	2.61	n.d.	3.48
		8	3.52	47.58	1.76	26.43	13.22	n.d.	n.d.	7.49
		9	3.68	9.47	12.11	61.05	7.37	n.d.	3.68	2.63
	17%	1	28.65	12.06	15.89	32.91	5.67	4.82	n.d.	n.d.
		2	23.55	13.64	17.28	39.47	4.68	1.38	n.d.	n.d.
		3	22.62	11.99	15.30	38.79	8.35	2.95	n.d.	n.d.
		4	20.98	11.48	14.39	42.08	6.80	4.28	n.d.	n.d.
		5	14.18	11.19	12.31	44.40	8.58	3.73	5.60	n.d.
		6	16.04	7.86	20.44	35.53	9.43	3.77	6.92	n.d.
		7	17.69	8.62	24.49	31.07	8.62	n.d.	6.58	2.95
		8	11.18	12.33	22.93	31.60	11.95	3.08	6.94	n.d.
		9	30.59	10.13	33.54	8.86	6.96	2.32	5.27	2.32
40 kPa	14%	1	14.11	5.93	30.88	16.97	20.04	n.d.	6.13	5.93
		2	18.80	4.41	30.74	20.34	15.04	n.d.	5.98	4.70
		3	20.18	3.68	19.09	33.69	14.92	n.d.	4.04	4.40
		4	16.32	4.20	17.73	40.41	12.46	n.d.	4.19	4.70
		5	18.93	4.27	8.27	34.67	15.73	n.d.	4.80	13.33
		6	23.16	3.86	7.37	43.51	13.33	n.d.	4.91	3.86
		7	40.38	11.97	9.62	15.81	10.47	n.d.	5.98	5.77
		8	35.71	10.08	8.61	18.49	10.29	n.d.	6.51	10.29
		9	22.79	6.05	49.30	n.d.	13.02	n.d.	5.58	3.26
	17%	1	24.54	10.44	15.75	21.79	10.44	5.86	5.31	5.86
		2	23.46	9.08	12.66	31.77	8.29	4.38	5.25	5.12
		3	22.93	8.99	9.34	32.36	7.42	4.37	4.35	10.25
		4	23.84	7.14	8.42	36.40	6.42	4.00	5.22	8.57
		5	30.25	4.37	4.54	41.18	4.37	5.71	5.55	4.03
		6	16.00	9.00	4.00	52.50	8.75	4.25	5.50	n.d.
		7	31.46	7.67	6.14	39.39	3.07	4.09	5.37	2.81
		8	39.48	6.26	6.26	30.09	8.70	n.d.	6.26	2.96
		9	27.64	6.10	45.12	10.98	6.10	n.d.	4.07	n.d.
80 kPa	14%	1	20.42	12.71	24.38	22.50	20.00	n.d.	n.d.	n.d.
		2	19.57	10.30	21.64	32.75	12.71	n.d.	n.d.	3.03
		3	20.01	8.85	22.11	33.50	11.89	n.d.	n.d.	3.64
		4	18.62	12.62	22.36	33.26	8.90	n.d.	n.d.	4.25
		5	16.34	15.05	23.79	33.50	6.47	n.d.	n.d.	4.85
		6	22.27	8.15	24.06	39.36	6.16	n.d.	n.d.	n.d.
		7	13.51	12.74	26.45	35.71	7.34	n.d.	n.d.	4.25
		8	11.71	15.73	25.00	37.41	6.82	n.d.	n.d.	3.32
		9	7.94	2.12	72.49	12.70	4.76	n.d.	n.d.	n.d.
	17%	1	35.24	6.58	7.22	17.20	19.75	7.01	7.01	n.d.
		2	35.69	6.32	5.86	20.11	13.39	13.19	n.d.	5.44
		3	35.87	6.35	5.36	19.94	15.46	11.67	n.d.	5.34
		4	36.69	5.67	5.30	19.97	16.55	10.59	n.d.	5.23
		5	29.04	4.53	8.92	25.07	18.56	4.11	5.67	4.11
		6	35.43	5.58	13.11	28.31	8.09	n.d.	5.72	3.77
		7	43.21	4.29	7.86	14.11	11.25	4.46	7.14	7.68
		8	40.28	10.22	7.66	12.77	13.75	4.13	7.27	3.93
		9	54.96	2.07	15.29	24.79	n.d.	1.65	1.24	n.d.

n.d.—not detected.

**Table 2:** Nutritional value in 100 g of corn groats.

Energy value	369 kcal/1582 kJ
Protein	7 g
Total fat	1.8 g
Saturated fatty acids	0.2 g
Monounsaturated fatty acids	0.3 g
Polyunsaturated fatty acids	0.6 g
Carbohydrates	79 g
Dietary fiber	3.9 g
Vitamins	
Vitamin A	214 I.U.
Vitamin E	0.12 mg
Vitamin B6	0.2 mg
Folic acid	85 μg
Pantothenic acid	0.848 mg
Minerals	
Calcium	8 mg
Iron	0.2 mg
Magnesium	114 mg
Phosphorus	285 mg
Potassium	195 mg
Sodium	5 mg
Zinc	1.68 mg
Copper	0.75 mg
Manganese	1.63 mg
Selenium	2.7 μg

## Data Availability

Data are contained within the article.

## Supplementary Materials

The following supporting information can be downloaded at: https://www.mdpi.com/article/10.3390/s24072187/s1, “01_Supplementary_Materials. Agrinose”; “02_Supplementary_materials_Volatile_compounds_”; “03_Supplementary_materials_An_electornic_nose_results”.
