# Supplementary material for: Application of MOX Sensors to Determine the Emission of Volatile Compounds in Corn Groats as a Function of Vertical Pressure in the Silo and Moisture Content of the Bed
Source: Sensors (Basel). 2024 Mar 28;24(7):2187. doi: 10.3390/s24072187 (PMC11014101; doi:10.3390/s24072187)
Supplement: Supplementary file 1 [file sensors-24-02187-s001.zip › 01_Supplementary_Materials. Agrinose_.pdf]

01_Supplementary Materials.

Electronic nose procedure

Agrinose (Food Volatile Compound Analyser) has been designed and built in the Institute of Agrophysics, PAS, Poland. The device consists of eight metal-oxide semiconductor (MOS) sensors, which were selected with the following criteria: lower power consumption, a similar type and geometry of the sensors in the array, low susceptibility to humidity and temperature, and common use of the gas sensors tested in a similar measurement device. As ensured by the producers, the sensors strongly respond to the presence of ketones, fatty acids, esters, and alcohols that can be expected in fungal metabolites in granular materials spoilage [7,21]. Table 1S presents the types and technical data of Agrinose sensors. Seven of them (TGS type) were produced by Figaro Engineering (Japan) and one by Ams (USA). A measurement cycle according to the sample protocol consisted of 10 s baseline purge, 100 s sample draw-in, 5 s laboratory air purge and 100 s sample purge. Analog signals were converted to digital signals by means of software DasyLab. Obtained sensorgrams were converted to the ∗.xls format and analysed using software Statistica (version 12.0, StatSoft Inc., USA).

Table 1S. Technical data of Agrinose sensors

Type	Description	Detecting range (ppm)
TGS2600 - B00	General air contaminants, hydrogen and carbon monoxide	1 - 3 (H_2)
TGS2610 - C00	LP gas, butane	500 - 10 000
TGS2602 - B00	Ammonia, Hydrogen sulfide (high sensitivity to VOC and odorous gases)	1 - 30 (EtOH)
TGS2611 - C00	Natural gas, methane	500 - 10 000
TGS2603 - B01	Odors generated from spoiled foods	1 - 10 (EtOH)
TGS2612 – D00	Methane, propane and butane	1-25%LEL
TGS2620 - C00	Solvent vapours, volatile vapors, alcohol	50 - 5 000
AS – MLV - P2	CO, butane, methane, ethanol, hydrogen. Specifically designed for volatile organic compounds (VOCs)	10 - 10 000
